# Minus end-directed kinesin-14 KIFC1 regulates the positioning and architecture of the Golgi apparatus

**DOI:** 10.18632/oncotarget.16863

**Published:** 2017-04-05

**Authors:** Zhen-Yu She, Meng-Ying Pan, Fu-Qing Tan, Wan-Xi Yang

**Affiliations:** ^1^ The Sperm Laboratory, College of Life Sciences, Zhejiang University, Hangzhou 310058, China; ^2^ The First Affiliated Hospital, College of Medicine, Zhejiang University, Hangzhou 310003, China

**Keywords:** kinesin-14, KIFC1, the Golgi apparatus, microtubule, the Golgi architecture

## Abstract

The Golgi apparatus is the central organelle along the eukaryotic secretory and endocytic pathway. In non-polarized mammalian cells, the Golgi complex is usually located proximal to the nucleus at the cell center and is closely associated with the microtubule organizing center. Microtubule networks are essential in the organization and central localization of the Golgi apparatus, but the molecular basis underlying these processes are poorly understood. Here we reveal that minus end-directed kinesin-14 KIFC1 proteins are required for the structural integrity and positioning of the Golgi complex in non-polarized mammalian cells. Remarkably, we found that the motor domain of kinesin-14 KIFC1 regulates the recognition and binding of the Golgi and KIFC1 also statically binds to the microtubules via its tail domain. These findings reveal a new stationary binding model that kinesin-14 KIFC1 proteins function as crosslinkers between the Golgi apparatus and the microtubules and contribute to the central positioning and structural maintenance of the Golgi apparatus.

## INTRODUCTION

The Golgi apparatus is the central organelle in the eukaryotic secretory and endocytic pathway. Though the Golgi apparatus is evolutionarily and functionally conserved, the shapes and numbers of the Golgi apparatus vary considerably between fungi, plants, invertebrates and mammals [1, 2]. In most mammalian cells, the Golgi complex is one-copy ribbon shaped organelle made up of several Golgi stacks connected by tubular networks [3–5]. In mammalian cells, the assembly process of the Golgi complex is complicated and can be roughly classified into three steps: membrane fusion and vesicle transport; the maintenance of the flattened stacks; and the formation of the characteristic Golgi ribbons [6].

The cytoskeletal networks, including the arrangement of the microtubules and actin filaments, are essential in the positioning and organization of the Golgi complex [7]. Rather than the roles of actin filaments in the early steps of formation and maintenance of the Golgi cisternae [8, 9], the microtubules are essential for the correct positioning and architectural maintenance of the Golgi ribbons at the pericentrosomal region at the cell center [10–13]. In non-polarized mammalian cells, the Golgi apparatus is closely associated with the microtubule organizing center (MTOC), which is also located next to the nucleus at the cell center [6, 14].

In non-polarized mammalian cells, γ-tubulin ring complexes (γ-TuRC) located around the centrosome nucleates the radial array of microtubules in which the minus ends are anchored at the centrosome and the plus ends extend towards the cell periphery [15]. The minus end-directed motor cytoplasmic dynein 1 has been highlighted as the primary motor mediating inward vesicle transport and Golgi positioning [16, 17]. Another minus end-directed kinesin-14 KIFC3 also participates in the Golgi integration and positioning but only under cholesterol-depleted conditions [18]. However, it is currently unclear whether other motor proteins are involved in the processes of localization and organization of the Golgi apparatus.

In this study, we reveal that the minus end-directed kinesin-14 KIFC1 is required for the positioning and organization of the Golgi apparatus in cultured cells. Remarkably, we find that the motor domain of kinesin-14 KIFC1 recognizes and binds to the Golgi apparatus and that the overexpression of the KIFC1 motor domain leads to Golgi dispersal and disorganization. Furthermore, we demonstrate that the targeted ablation of KIFC1 results in a disrupted positioning of the Golgi apparatus and the dispersal of the Golgi apparatus into mini-stacks or cisternae which are observed as scatters throughout the cytoplasm. Taken together, we find that the kinesin-14 KIFC1 serves as the crosslinker between the Golgi apparatus and the microtubules around the centrosome, where the KIFC1 tail domain anchors to the microtubules and the motor domain binds to the Golgi apparatus. These results could shed a new light on the molecular basis underlying the positioning and structural maintenance of the Golgi apparatus in non-polarized mammalian cells.

## RESULTS

### Kinesin-14 KIFC1 can partially co-localize with the Golgi apparatus in cultured cells

**Figure 1:**
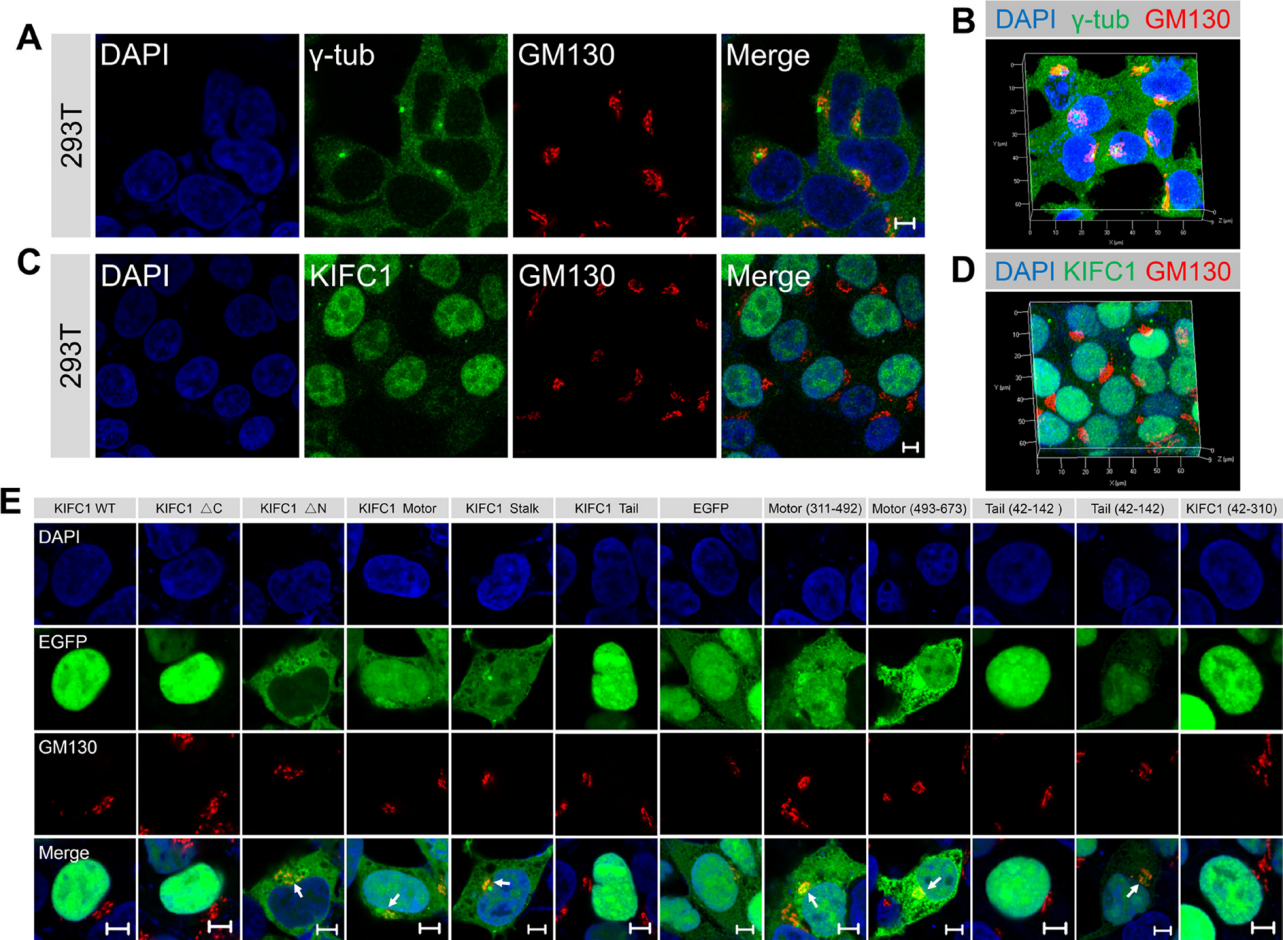
KIFC1 accumulates at the Golgi apparatus at cultured HEK293T cells See also Supplementary Figures 1, 2. (**A**) Representative confocal images of γ-tubulin and the Golgi marker GM130 in HEK293T cells. DAPI, blue; γ-tubulin, green; GM130, red. Scale bars, 5 μm. (**B**) Three dimensional images of the fluorescence signal of nucleus (blue), γ-tubulin (green) and the Golgi marker GM130 (red) in HEK293T cells. (**C**) Representative immunofluorescence images of KIFC1 and the Golgi marker GM130 in HEK293T cells. DAPI (blue); KIFC1 (green); GM130 (red). Scale bars, 5 μm. (**D**) Three dimensional images of the fluorescence signal of nucleus (blue), KIFC1 (green) and the Golgi marker GM130 (red) in HEK293T cells. (**E**) HEK293T cells were transiently transfected with KIFC1 WT (wild type, 1-673), KIFC1 ΔC, KIFC1 ΔN, KIFC1 Motor, KIFC1 Stalk, KIFC1 Tail, EGFP, Motor (311-492), Motor (493-673), Tail (42-142), Tail (42-310), KIFC1 (42-310) for 24 hr. DAPI, blue; EGFP fusion proteins, green; GM130, red. Scale bars, 5 μm.

To determine whether the kinesin-14 KIFC1 proteins are enriched at the Golgi apparatus, indirect immunofluorescence assays were used to examine the localization of KIFC1 proteins in two non-polarized mammalian cell lines, HEK293T cells and HeLa cells. We examined the MTOC using a γ-tubulin antibody with a co-stain containing the Golgi marker GM130 in both HEK293T and HeLa cells. We confirmed that the MTOC was indeed located near the nucleus and that the Golgi apparatus exhibited a characteristic tubular network which was located around the MTOC in these two cell lines (Figure 1A, 1B; Supplementary Figure 1A, 1B). In these two non-polarized cells, the Golgi apparatus is a single-copy ribbon-like organelle consisting of several flat stacks joined by a continuous tubular network and located closely to the nucleus at the cell center. We found that a portion of the KIFC1 proteins were co-localized with the Golgi marker protein GM130, and that KIFC1 proteins were partially located at the Golgi apparatus in HEK293T and HeLa cells at interphase (Figure 1C and Supplementary Figure 1E). We quantified fluorescence intensities via a line scan plot using Image J software, and we found that the intensities of a portion of KIFC1 proteins and GM130 were correlated at the Golgi apparatus near the nucleus and that the peak area of these two fluorescence signals was at almost the same locations (Supplementary Figure 1C, 1D). Using confocal laser scanning microscopy, we also examined the localization of KIFC1 at the Golgi apparatus at a three dimensional level. The KIFC1 proteins were accumulated at the Golgi apparatus at the perinuclear region and the Golgi apparatus was located and identified as closely associated with the nucleus at the cell center (Figure 1D). In addition, the specificity of the KIFC1 antibodies were tested in the immunofluorescence assays (Supplementary Figures 1F and 4C) where this was also examined by western blotting assay (Supplementary Figures 1G and 4B). The specificity of the KIFC1 antibodies fulfill the requirements in this study.

### The motor domain of kinesin-14 KIFC1 recognizes and partially binds to the Golgi apparatus

Kinesin-14 KIFC1 is comprised of three functional domains: the N-terminal tail domain, the coiled-coil stalk domain and the C-terminal motor domain. To determine which domain is required for the accumulation of KIFC1 proteins at the Golgi apparatus, we initially generated EGFP-tagged fusion constructs: the KIFC1 WT (wild type; full length), KIFC1 ΔC, KIFC1 ΔN, KIFC1 Motor, KIFC1 Stalk, KIFC1 Tail, EGFP control constructs (Supplementary Figure 2A) and transfected these into HEK293T cells that were then cultured for 24 hours (Supplementary Figure 2D and Supplementary Figure 3).

We found that the full-length KIFC1-EGFP proteins were mostly enriched at the nucleus, and only a small proportion were distributed throughout the cytoplasm (Figure 1E). Similarly, KIFC1 ΔC-EGFP proteins were largely located in the nucleus (Figure 1E). The distribution of KIFC1 Tail-EGFP proteins showed similar patterns with the KIFC1 WT and KIFC1 ΔC (Figure 1E). More strikingly, we found that both KIFC1 ΔN and KIFC1 Motor-EGFP fusion proteins largely accumulated at the Golgi apparatus and highly overlapped with the GM130 fluorescence signals at the Golgi region (Figure 1E). A small portion of the KIFC1 Stalk-EGFP proteins were also located at the Golgi apparatus, but to a lesser proportion and intensity than the previous examples (Figure 1E and Supplementary Figure 2F). In the control group, EGFP alone exhibited a relatively wide and non-specific distribution throughout the cytoplasm and nucleus, displaying little specific accumulation at the Golgi apparatus and the nucleus (Figures 1E and Supplementary Figure 2F). Taken together the above data suggest that a large portion of the motor domain of KIFC1 can recognize and bind to the Golgi apparatus.

To further investigate which parts of the motor domain are required for KIFC1 proteins partial accumulation at the Golgi apparatus, we transiently expressed Motor (311-492) and Motor (493-673)-EGFP fusion proteins in HEK293T cells (Supplementary Figure 2B). We found that a majority of these fusion proteins were localized at the Golgi apparatus and Motor (493-673)-EGFP could bind to the Golgi apparatus more efficiently than other fusion proteins and were largely enriched at the Golgi apparatus (Figure 1E, Supplementary Figure 2B and 2F).

We next examined whether the tail domain of KIFC1 could target the Golgi apparatus. We considered that the nuclear localization signal (NLS) at the N-terminal of KIFC1 proteins may transport KIFC1 proteins into the nucleus rather than into the cytoplasm or the Golgi apparatus. The NCBI GenBank database suggests that in humans, KIFC1 proteins may have several different isoforms. Differences between such isoforms can include differences in the beginnings of the open reading frames between different genes. Due to such isoform variations occurring in the initial 1–41 amino acids, we therefore constructed the Tail (42-142) and KIFC1 (42-310)-the EGFP fusion proteins and expressed each of these separately in HEK3293T cells for 24 hr. We found that Tail (42-142) and KIFC1 (42-310)-EGFP proteins mostly remained in the nucleus (Figure 1E, Supplementary Figure 2C, 2E). However, in 17.0 % cells, a portion of the Tail (42-142)-EGFP fusion proteins have been transported out from the nucleus to the cytoplasm, where they exhibited a localization pattern around the Golgi (Figure 1E and Supplementary Figure 2F). The likely explanation for this phenomenon is that for KIFC1 proteins the classical nuclear localization signal, NLS; 36-51AAs; KRRPDQMEDGLEPEKK, is partitioned into two parts and the resulting defects in nuclear transport lead to the observed cytoplasmic accumulation. These results suggest that in the absence of the N-terminal 1–41 amino acids, a portion of the KIFC1 tail domain can also target the Golgi apparatus.

Finally, we quantified the localization patterns of each domain of KIFC1 at the Golgi apparatus, in the cytosol and at the nucleus, we found that the KIFC1 ΔN, KIFC1 Motor, Motor (311-492) and Motor (493-673)-EGFP fusion proteins mainly localized at the Golgi apparatus and the cytosol, whereas the KIFC1 Tail and KIFC1 Tail (42-142) are mainly localized at the nucleus (Supplementary Figure 2E). Taken together, the above data demonstrate that the KIFC1 motor domain can efficiently target the Golgi apparatus and that a portion of the tail domain can also recognize the Golgi apparatus in the absence of the N-terminal 1–41 amino acids. This indicates that KIFC1 proteins can bind to the Golgi in two independent ways via the mediation of either the C-terminal motor domain or the N-terminal tail domain.

### Dominant negative effects of the KIFC1 motor domain on the positioning and architecture of the Golgi apparatus

**Figure 2:**
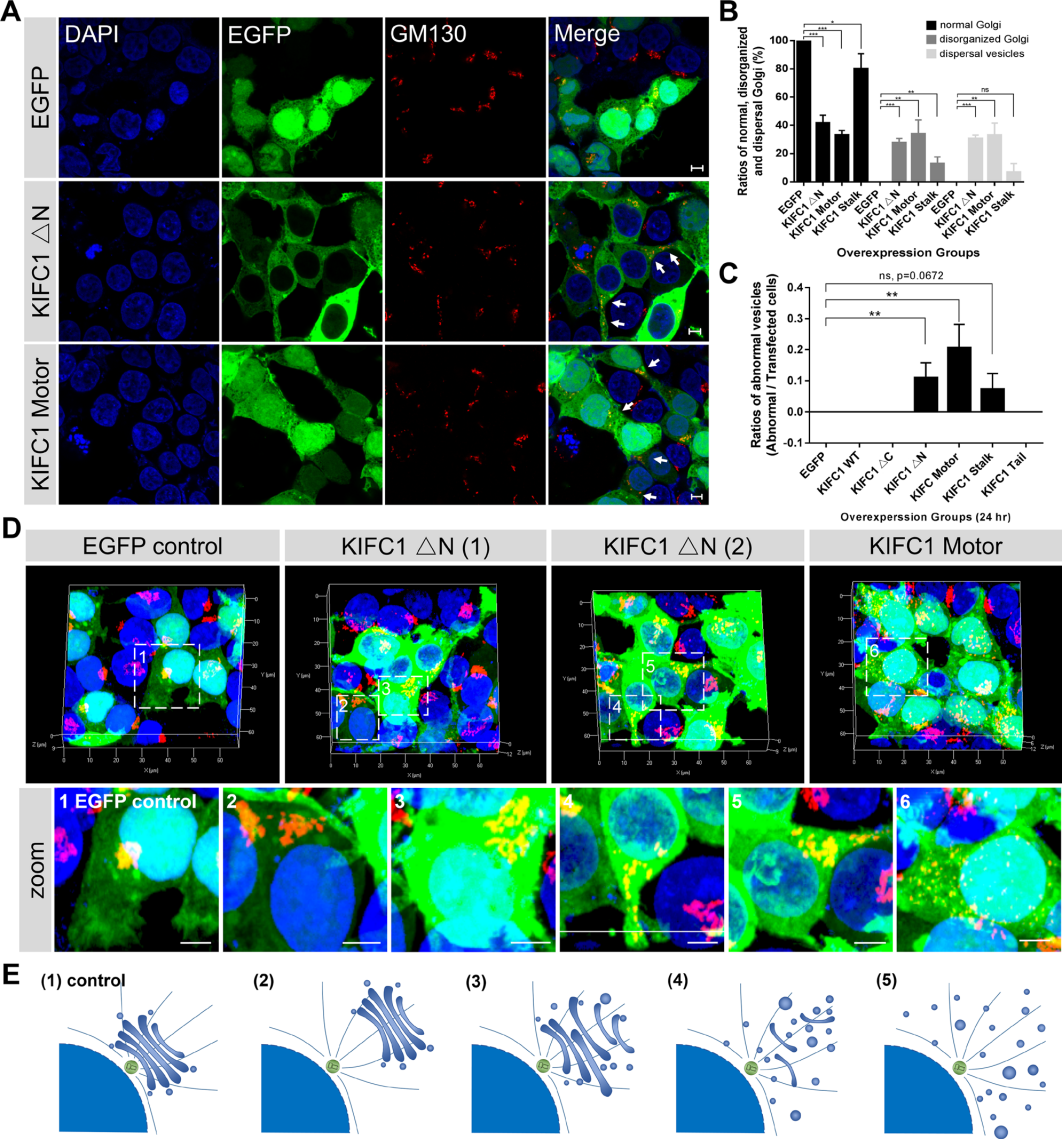
Dominant negative effects of KIFC1 ΔN and KIFC1 motor constructs on the Golgi apparatus See also Supplementary Figures 3, 4. (**A**) Representative confocal images shows the nucleus (DAPI), KIFC1 ΔN and KIFC1 motor EGFP fusion constructs (green) and the Golgi apparatus (GM130). Scale bars, 5 μm. (**B**) Quantification the normal Golgi, disorganized Golgi and scattered Golgi-associated vesicles in the transfected HEK293T cells after overexpression with EGFP control, KIFC1 ΔN, KIFC1 Motor and KIFC1 Stalk for 24 hr, respectively. Data are represented as mean ± SEM. (**C**) Quantification of the abnormal EGFP-accumulated vesicles in the transfected HEK293T cells after overexpression with each KIFC1 mutant construct, respectively, for 24 hr. Data are represented as mean ± SEM. (**D**) Three dimensional images of KIFC1 ΔN and KIFC1 motor constructs in HEK293T cells. DAPI (blue), EGFP fusion proteins (green), GM130 (red). The zoom indicates the magnified view of the outlined boxes. Scale bars, 5 μm. (**E**) (1) In the control, the Golgi apparatus is a single-copy ribbon-like organelle, which is closely associated with the centrosome near the nucleus. Overexpression of the KIFC1 ΔN and KIFC1 motor constructs results in several defects: (2) The Golgi apparatus seems normal, but located away from the nucleus; (3-4) The characteristic stacked Golgi morphology is aberrant and several Golgi cisterna are dispersed at the cytosol; (5) Many Golgi mini-stacks and cisternae are distributed throughout the cytoplasm.

Surprisingly, we noticed that after 24 hours overexpression of KIFC1 Motor and KIFC1 ΔN-EGFP fusion proteins, the structures of the Golgi apparatus became disrupted in most transfected cells. In contrast to the regular and well-organized structures of the wild type in the EGFP overexpression group, both the KIFC1 Motor and KIFC1 ΔN overexpression resulted in the disrupted localization of the Golgi apparatus and led to the fragmentation of the Golgi apparatus into disorganized Golgi-associated cisternae and mini-stacks, and even an accumulation of large vesicles (Figure 2A–2C; Supplementary Movies 3–8). Quantification of the ratios of the disorganized Golgi apparatus in the transfected HEK293T cells showed that 33% of the KIFC1 Motor and 27% of the KIFC1 ΔN mutants transfected cells were disrupted in the Golgi localization (Figure 2B). And 33% of the KIFC1 Motor and 30% of the KIFC1 ΔN mutants transfected cells exhibited dispersal of Golgi-associated vesicles and mini-stacks (Figure 2B). This suggests that the KIFC1 Motor and KIFC1 ΔN mutant proteins exert dominant effects on the Golgi apparatus, including the disruption of central Golgi positioning and the dispersal of the Golgi vesicles and mini-stacks.

Further analysis of the Golgi apparatus was conducted for the cultured HEK293T cells, where we examined the overall architecture of the Golgi apparatus in the overexpression of KIFC1 Motor and KIFC1 ΔN mutants using three dimensional confocal microscopy (Figure 2D). We found that defects of the Golgi apparatus could be roughly divided into four subfamilies, depending on aspects of localization and other structural changes in the Golgi apparatus (Figure 2E): (i) The structure of the Golgi apparatus was intact, but lost its perinuclear localization; (ii) the Golgi apparatus has become loose and disorganized and was disrupted slightly in both localization and structure; (iii) the Golgi apparatus was partially fragmented into peripheral mini-stacks and where several Golgi ribbon remained in the cytoplasm; and (iiii) where the Golgi apparatus has become completely dispersed into peripheral mini-stacks and vesicles throughout the cytoplasm.

We next examined whether other functional domain mutant proteins could exert similar dominant negative effects on the Golgi apparatus. We found that the overexpression of KIFC1 Stalk-EGFP resulted in a minor effects in the Golgi apparatus in several cells (12% disorganized and 6% dispersal mini-stacks and vesicles) where the Golgi ribbons were slightly influenced in their localization and organization (Figure 2B, Supplementary Figure 4A, 4B). As expected, the overexpression of Motor (311-492) and Motor (493-673) mutants resulted in similar effects on the Golgi apparatus as observed in the KIFC1 motor domain mutants (Supplementary Figure 3A, 3B). In contrast, the overexpression of the Tail domain, Tail (42-142) and KIFC1 (42-310) mutants did not have an obvious influence on the Golgi apparatus. Taken together, we confirmed that the KIFC1 Motor and the KIFC1 ΔN mutant proteins exert dominant negative effects on the Golgi apparatus which result in the dispersal and disorganization of the Golgi apparatus.

### KIFC1 ablation results in the Golgi apparatus dispersal and disorganization

**Figure 3:**
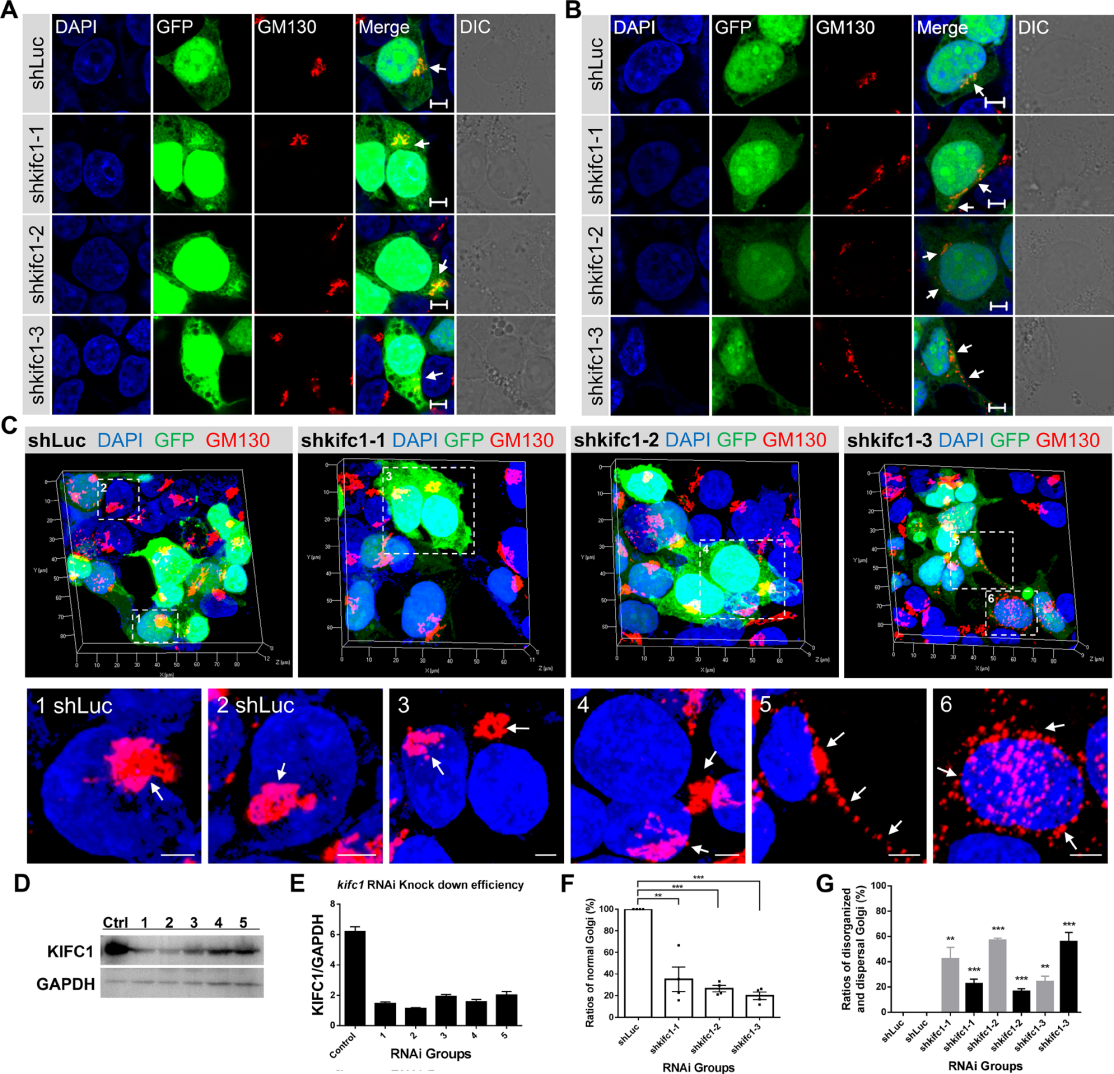
Depletion of KIFC1 led to the disorganization and dispersal of the Golgi apparatus (**A**, **B**) HEK293T cells were transfected with the shLuc, shkifc1-1, shkifc1-2, shkifc1-3 shRNA plasmids for 90 hr and analyzed by immunofluorescence assays, respectively. shLuciferase was used for the control group. DAPI (blue), GFP (green), GM130 (red), DIC (Differential Interference Contrast). Scale bars, 5 μm. (**C**) Representative three dimensional images of the Golgi apparatus (GM130, red) in the shRNA transfected HEK293T cells. The zoom is the magnified view of the indicated box. Scale bars, 5 μm. (**D**, **E**) Western Blot analysis of RNAi knockdown efficiency in HEK293T cells after 90 hr. Five different *kifc1* targeting shRNA plasmids were used as candidates. The first, second and fourth plasmid (1, 2, 4 designated as shkifc1-1, shkifc1-2 and shkifc1-3, respectively) were selected for this study. GAPDH was used as the loading control. (**F**) Quantification the ratios of cells containing normal Golgi apparatus in the transfected HEK293T cells after *kifc1* knockdown assay for 90 hr. Data are represented as mean ± SEM. ns, not significant; *P < 0.05; **P < 0.01; ***P < 0.001 (Student's *t*-test). (**G**) Quantification of the ratios of disorganized Golgi apparatus (gray color) and the dispersed Golgi (black color) into vesicles after s after *kifc1* knockdown assay for 90 hr. Data are represented as mean ± SEM. ns, not significant; *P < 0.05; **P < 0.01; ***P < 0.001 (Student's *t*-test).

Based on the dominant negative effects of the KIFC1 motor domain on the localization and organization of the Golgi apparatus, we wanted to see if the KIFC1 knock down could also result in similar phenotypes. We used five shRNA expression constructs to knockdown the endogenous KIFC1 in HEK293T cells. We then selected three efficient target shRNA constructs as the experimental subjects (designated as shkifc1-1, shkifc1-2 and shkifc1-3) (Figure 3A–3G). Consistent with previous dominant negative effects, we found that KIFC1 knockdown could also lead to the disrupted localization of the Golgi apparatus (Figure 3A) and dispersal of the Golgi apparatus into small mini-stacks and vesicles throughout the cytoplasm (Figure 3B).

**Figure 4:**
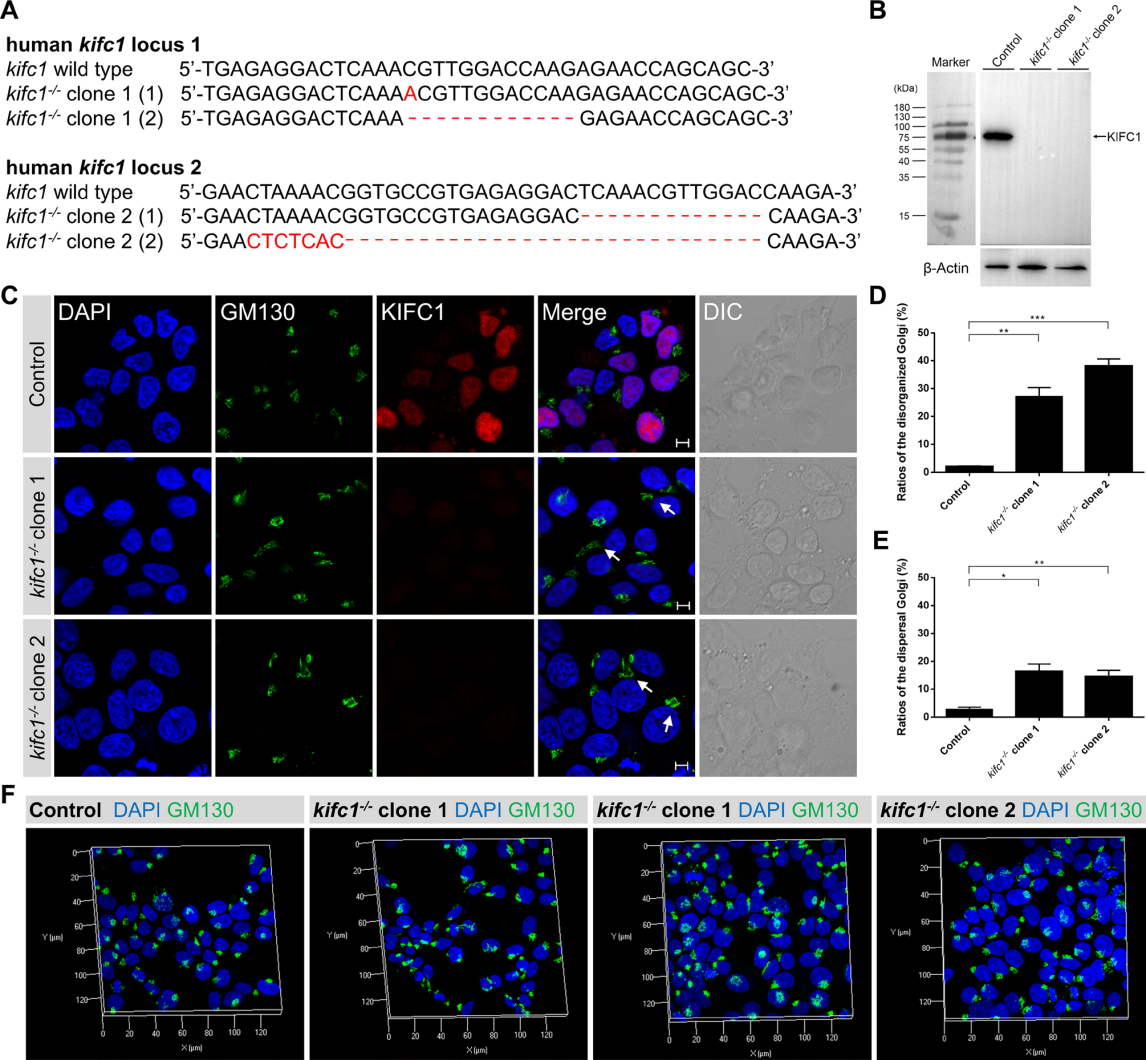
*Kifc1* knockout also results in the disorganization and dispersal of the Golgi apparatus (**A**) Representative sequences of the human *kifc1* locus targeted by Cas9n and the selected sequences showing representative indels. Generation of indels in the human *kifc1* locus in HEK293T cells by CRISPR-Cas9 system. Two different *kifc1* knockout cell lines were selected as candidates in this study (designated as *kifc1*^−/−^ clone 1 and clone 2). (**B**) Western blot analysis of KIFC1 expression in control, *kifc1^−/−^* clone 1 and clone 2 HEK293T cells. β-Actin was used as the loading control. (**C**) Representative confocal images of the Golgi marker GM130 and KIFC1 in control and *kifc1* knockout HEK293T cells (*kifc1^−/−^* clone 1 and clone 2). DAPI, blue; GM130, green; KIFC1, red. DIC, differential interference contrast. Scale bars: 5 μm. (**D**) Quantification of the ratios of disorganized Golgi apparatus in control and *kifc1* knockout HEK293T cells (*n* = 3 cultures for each phenotype). Data are presented as means ± SEM. ns, not significant; *P < 0.05; **P < 0.01; ***P < 0.001 (Student's *t*-test). (**E**) Quantification of the ratios of the dispersal Golgi in control and *kifc1* knockout HEK293T cells (*n* = 3 cultures for each phenotype). Data are presented as means ± SEM. ns, not significant; *P < 0.05; **P < 0.01; ***P < 0.001 (Student's *t*-test). (**F**) Representative three dimensional images of the Golgi apparatus (DAPI, blue; GM130, green) in the control, *kifc1^−/−^* clone 1 and clone 2 HEK293T cells.

Furthermore, we also utilized the CRISPR-Cas9 genome editing approaches to knockout the human *kifc1* gene in HEK293T cells (Figure 4A, 4B). We then selected two different *kifc1^−/−^* HEK293T cell lines as experimental candidates (designated as *kifc1^−/−^* clone 1 and *kifc1^−/−^* clone 2). Consistent with the *kifc1* gene knockdown assays, we revealed that the completely gene knock out of *kifc1* gene in HEK293T cells resulted in the disorganization of the Golgi apparatus and the dispersal of the Golgi into cisternae and vesicles (Figure 4A–4F).

Consistent with our previous results, the use of three dimensional microscopy revealed that, in several KIFC1 ablation cells, the Golgi apparatus was disassembled and was located a significant distance from the perinuclear area (Figures 3C and 4F). In other KIFC1 depletion cells, the elements of the Golgi apparatus had been dispersed into mini-stacks and small cisternae appeared to be scattered throughout the cytoplasm (Figures 3C and 4F). This demonstrates that the kinesin-14 KIFC1 protein is required for the localization of the Golgi apparatus closely proximal to the nucleus at the cell center and is essential to the organization and interconnection of the components of the Golgi apparatus in HEK293T cells.

The *kifc1* knockdown and knockout efficiency was examined using a western blot assay (Figure 3D, 3E; Figure 4B). To quantitatively evaluate the effects of KIFC1 knockdown on the Golgi apparatus we classified the different phenotypes into two groups (disorganized Golgi and dispersal of vesicles) according to the localization and structure of the Golgi apparatus (Figure 3F, 3H). We observed that 42.2%, 56.8% and 24.2% of the shkifc1-1, 2 and 3 transfected cells were affected, respectively, in which the elements of the Golgi apparatus had been disassociated and transported away from the nucleus being unable to stay close to the perinuclear region (Figure 3G). Similarly, we found that 2.1%, 27.2% and 38.1% of the control, *kifc1^−/−^* clone 1 and *kifc1^−/−^* clone 2 HEK293T cells exhibited the disorganized Golgi apparatus (Figure 4D). We also found that 22.7%, 16.6% and 55.8% of the shkifc1-1, 2 and 3 transfected cells exhibited the phenotype that the Golgi apparatus dispersed into mini-stacks scattering throughout the cytoplasm (Figure 3G). In addition, 2.8%, 16.4% and 14.5% the control, *kifc1^−/−^* clone 1 and *kifc1^−/−^* clone 2 HEK293T cells exhibited the dispersed Golgi apparatus (Figure 4E).

### Dynamics of minus end-directed kinesin-14 KIFC1 proteins at the Golgi apparatus

To examine how KIFC1 proteins stimulate the localization and organization of the Golgi apparatus, we used time-lapse microscopy and tracked the movements of KIFC1-EGFP and other mutant EGFP fusion proteins in HEK293T cells. We found that the KIFC1 WT-EGFP proteins were highly dynamic in both the cytoplasm and the nucleus in HEK293T cells and that KIFC1 WT-EGFP proteins were observed to move to the minus ends at the MTOC along the spindles during mitosis (Supplementary Movie 1). KIFC1 ΔC-EGFP fusion proteins were accumulated mainly in the nucleus of HEK293T cells and moved in a similar pattern to that of KIFC1 WT (Supplementary Movie 2). In addition, KIFC1 Tail-EGFP were also enriched in the nucleus, but with a low motility (Supplementary Movie 6). Consistent with our previous results, this findings suggest that the tail domain of KIFC1 results mainly in the nuclear accumulation of KIFC1 proteins and that full length wild-type KIFC1 proteins represents dynamic molecular motors in both the nucleus and the cytoplasm.

**Figure 5:**
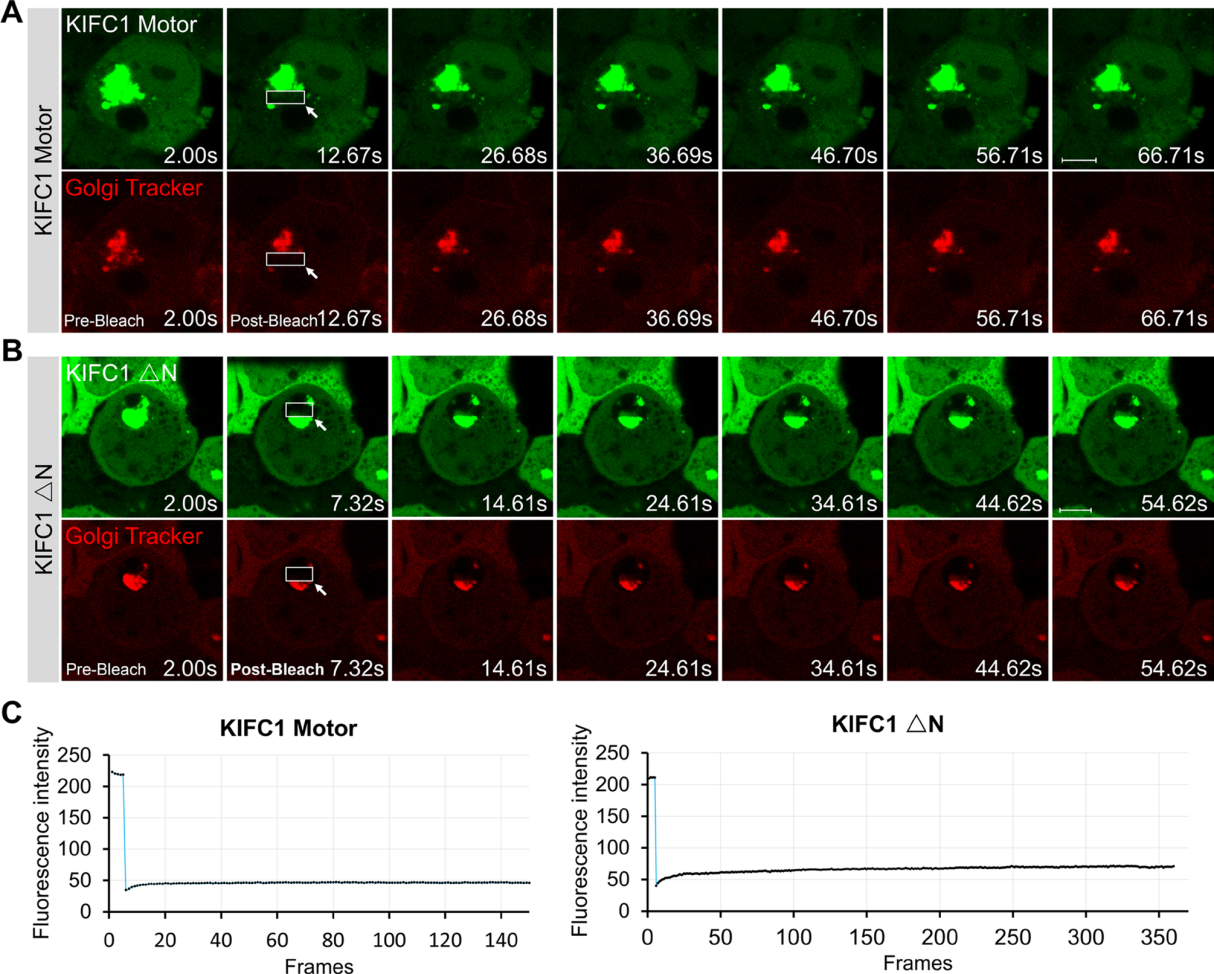
Dynamic of KIFC1 motor and KIFC1 ΔN EGFP-fusion proteins (**A**, **B**) Time-lapse images of KIFC1 Motor (A) and KIFC1 ΔN (B) EGFP fusion proteins expressed in the HEK293T cells for 24 hr. Before live-cell imaging, cell were stained with the Golgi Tracker (red). The indicated boxes are the photo-bleaching areas. The fluorescence in this area is photo-bleach by a strong 405 nm laser using CLSM710 microscope (Zeiss) at the beginning of the experiments. The images were collected using CLSM710 microscope every 500 ms. The representative images of the fluorescence recovery in the FRAP experiments were shown in Figure 5A and 5B. The time for each figure was shown at bottom right. Scale bars, 5 μm. (**C**) Quantification of the recovery rate the KIFC1 motor (left panel) and KIFC1 ΔN (right panel) in the indicated photo-bleaching area using Image J software. The Y axis shows the fluorescent intensity of EGFP signals in the indicated box; The X shows the frames, 500 ms/frames.

In this study, we observed that the KIFC1 ΔN-EGFP and KIFC1 Motor-EGFP fusion proteins were largely located in the cytoplasm. Remarkably, consistent with our dominant negative effect experiments, we found that a portion of the cells that displayed the overexpression of these two KIFC1 mutants exhibited aberrant accumulations of vesicles in HEK293T cells (Figure 2C; Figure 5A, 5B; Supplementary Movies 3 and 4). The KIFC1 Motor-EGFP group showed a more severe phenotype (20.7%) than the KIFC1 ΔN-EGFP group (11.0%). A similar phenotype was also seen occasionally in the KIFC1 Stalk group (7.3%) (Figure 2C; Figure 5A, 5B; Supplementary Movie 5). This data indicates that the motor domain of KIFC1 can act to interrupt vesicle transport resulting in the aberrant accumulations of large vesicles.

To further investigate the dynamics of the KIFC1 motor domain and KIFC1 ΔN-EGFP in HEK293T cells, we used a fluorescence recovery after photobleaching (FRAP) technique to study their motility in the aberrant vesicles. We observed that the Golgi-Tracker co-stained with the EGFP fusion proteins in the aberrant vesicles. We also found that the KIFC1 Motor and KIFC1 ΔN-EGFP were also co-located at many small vesicles in the cytoplasm and displayed dynamic movement patterns (Figure 5A, 5B; Supplementary Movies 7 and 8). However, the larger vesicles remained static in the cytoplasm and the quantifications of the recovery rates of KIFC1 Motor and KIFC1 ΔN-EGFP in such aberrant vesicles indicated that neither of these two constructs showed any dynamic behavior (Figure 5C). Our study suggests that KIFC1 motor and KIFC1 ΔN can recognize and bind to small motile vesicles in the cytoplasm but also that overexpression of these two mutants also results in the accumulation of several large, static and aberrant vesicles in the cytoplasm.

### The interactions between KIFC1 and microtubules for the positioning and structural maintenance of the Golgi apparatus

**Figure 6:**
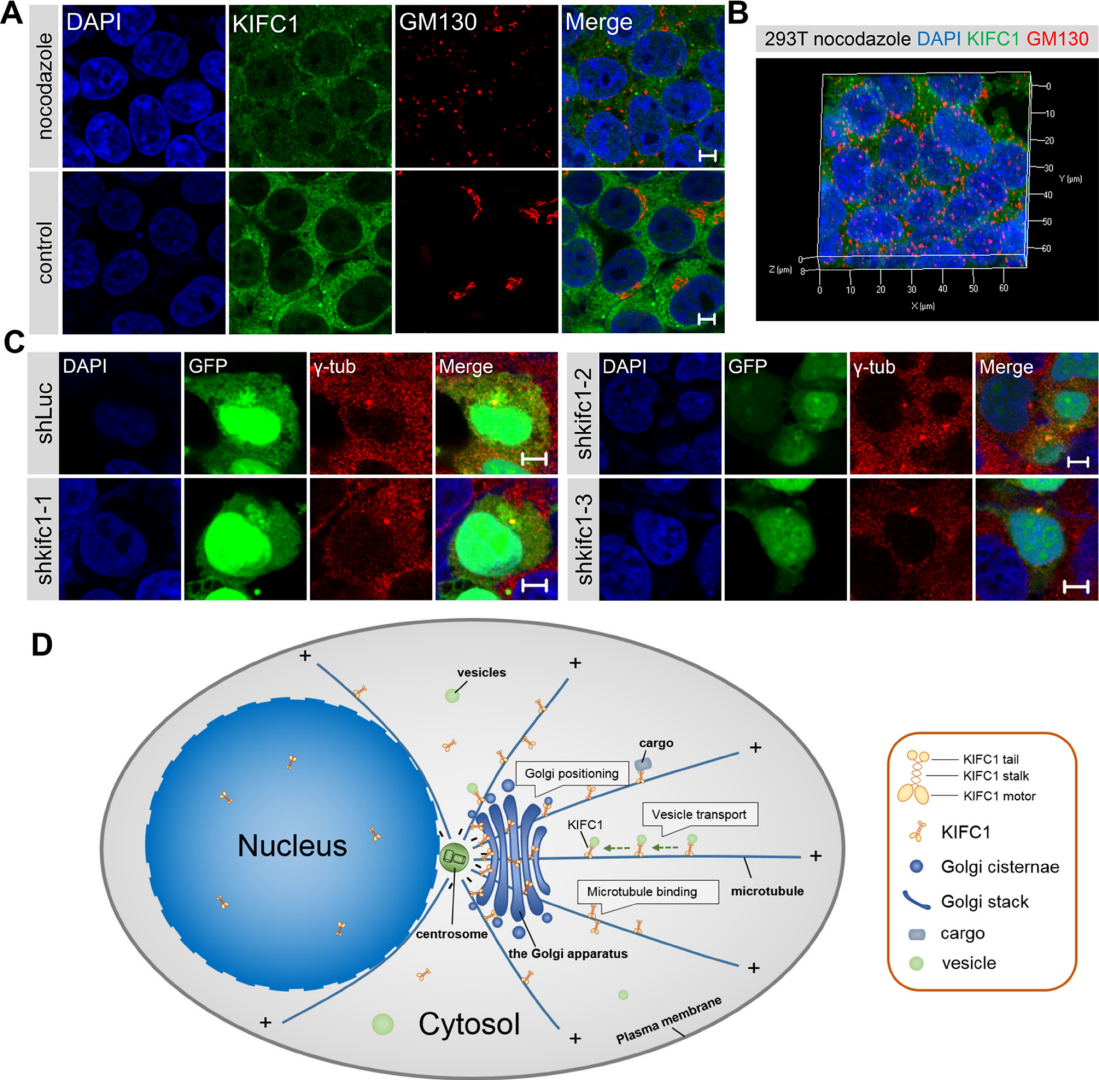
The interactions between kinesin-14 KIFC1 and microtubule networks in the Golgi positioning (**A**) Representative immunoflurescence images of KIFC1 and the Golgi marker GM130 in HEK293T cells in the control cells and in the nocodazole treatment group (10 μM/3 h). DAPI (blue), KIFC1 (green), GM130 (red). Scale bars, 5 μm. (**B**) Three dimensional images of the fluorescence signal of nucleus (blue), KIFC1 (green) and the Golgi marker GM130 (red) in HEK293T cells after incubation with nocodazole (10 μM/3 h). (**C**) Representative images of the localization of γ-tubulin in HEK293T cells after *kifc1* knockdown for 72 hr. DAPI (blue), GFP (green), γ-tubulin (red). GFP is the fluorescence protein in shRNA plasmid, and is used to indicate the transfection of shRNA plasmid. (**D**) The model for the functions of the minus end-directed kinesin-14 KIFC1 in the Golgi positioning and architectural maintenance in non-polarized mammalian cells. The microtubules are organized at the MTOC near the cell center and form characteristic radial arrays extending toward the cell periphery in non-polarized mammalian cells. In the cytoplasm, kinesin-14 KIFC1 proteins traffic along the microtubules, from the plus ends to the minus ends, and accumulate at the pericentrosomal region. During vesicle transport, kinesin-14 KIFC1 transports the Golgi-associated vesicles to the minus ends near the MTOC. The dynamic movements of kineisn-14 KIFC1 motors along the microtubules are fulfilled by the step-by-step walking of the motor domain using ATP-hydrolyzing energy and the cargo-binding affinity of the tail domain. Moreover, at the minus end of the microtubules around the MTOC, the kinesin-14 KIFC1 protein can bind to the Golgi apparatus via its motor domain and can also statically bind to the microtubules via its tail domain, thus severing as a static crosslinker between the Golgi apparatus and the microtubules around the pericentrosomal region near the nucleus.

What are the roles of microtubules in the Golgi positioning and architectural maintenance in non-polarized mammalian cells? To address this question, we examined the morphology and positioning of the Golgi apparatus in HEK293T cells in the absence and presence of nocodazole. Microtubule depolymerization by nocodazole (10 μM/3h) in HEK293T cells resulted in the characteristic ribbon-like Golgi apparatus which, whilst being located near the nucleus in the wild type, were dispersed into numerous Golgi-associated vesicles and mini-stacks which were scattered throughout the cytoplasm (Figure 6A and 6B). Our data suggest that the microtubules are essential to the architectural maintenance of the Golgi apparatus and its positioning near the nucleus in non-polarized mammalian cells.

We then turned to the question of whether the Golgi apparatus closely associates with the microtubule organizing center (MTOC), which is also located near the nucleus in non-polarized mammalian cells. We examined the MTOC using a γ-tubulin antibody with a co-stain containing the Golgi marker GM130 in both HEK293T and HeLa cells. We confirmed that the MTOC was indeed located near the nucleus and that the Golgi apparatus exhibited a characteristic tubular network which was located around the MTOC in these two cell lines (Figure 1A and Supplementary Figure 1A). This close association between the Golgi apparatus and the MTOC occurring near the nucleus suggests that the MTOC may be a crucial factor in the Golgi apparatus positioning and integrity.

We also examined whether the targeted depletion of KIFC1 in cultured cells influenced the positioning of the MTOC near the nucleus at the cell center. Similarly, we also observed the positioning of the centrosome in the normal and KIFC1 knockdown HEK293T groups. We found that compared to the control group, the knockdown of KIFC1 in HEK293T cells did not influence the central positioning of MTOC near the nucleus (Figure 6C). We concluded that the minus end-directed kinesin-14 KIFC1 proteins do not determine or influence the positioning of MTOC in HEK293T cells.

## DISCUSSION

Our findings revealed that the minus end-directed kinesin-14 KIFC1 proteins are necessary for the positioning and the structural integrity of the Golgi apparatus in non-polarized mammalian cells. Remarkably, we found that overexpression of the KIFC1 motor domain proteins results in the Golgi apparatus becoming dispersed and scattered throughout the cytoplasm. Consistent with these results, the KIFC1 knockdown also led to a similar disrupted localization of the Golgi apparatus and the dispersal of the Golgi apparatus into mini-stacks. Together, these results suggest that kinesin-14 KIFC1 proteins mediate the localization and organization of the Golgi apparatus at the cell center in non-polarized mammalian cells.

In this study, we found that both Motor (311-492) and Motor (493-673)-EGFP fusion proteins can accumulated at the Golgi apparatus. This indicates that both parts of motor domain of KIFC1 can recognize and bind to the Golgi apparatus. But which exact motifs in the motor domain are required for binding to the Golgi apparatus need to be further explored in future study. In this study, the motor domain of KIFC1 acts to interrupt vesicle transport resulting in the aberrant accumulations of large vesicles. The KIFC1 Motor domain mutant constructs may interact with the binding sites for normal KIFC1 proteins in a competitive manner, and thus results in the loss-of-function of normal KIFC1 proteins and defects in vesicle transport mediated by minus-end directed motility of KIFC1 proteins.

### The roles of microtubules in the positioning and structural maintenance of the Golgi

The Golgi apparatus is closely associated with the MTOC and located proximal to the nucleus at the center of non-polarized mammalian cells [6, 14, 19, 20]. Such Golgi positioning is essential for aspects of polarized secretion, cell migration, immunological synapse formation and axon specification [21]. Our study revealed that it was the microtubules which were particularly crucial for the Golgi positioning and architectural maintenance and that the Golgi apparatus was indeed closely associated with the MTOC near the nucleus in HEK293T cells and HeLa cells. Moreover, the knockdown of KIFC1 in HEK293T cells resulted in the Golgi apparatus becoming dispersed into the cytoplasm where it was no longer located close to the nucleus and had become disorganized into scattered Golgi mini-stacks. However, such a knockdown did not influence the central localization of MTOC near the nucleus. These observations raised the question of how interactions between kinesin-14 KIFC1 and microtubules influence the positioning and integrity of the Golgi apparatus.

In our present study, HEK293T cells treated with nocodazole (10 μM/3 h) resulted in the complete dispersal of the Golgi apparatus into numerous Golgi mini-stacks and Golgi-associated vesicles which were observed to be uniformly scattered throughout the cytoplasm. Consistent with previous studies [11, 12, 22], our data demonstrate that the microtubule network is essential in the positioning and organization of the Golgi apparatus in non-polarized mammalian cells.

### The functions of kinesin motors and dynein in the Golgi positioning and ER-to-Golgi transport

The functions of microtubules are hard to imagine in the Golgi positioning and integrity without considering the molecular motors. Molecular motors are considered to be the most important factors in the microtubule-dependent Golgi positioning [23, 24]. Cytoplasmic dynein 1 is the most well-established molecular motor for the minus end-directed transport of Golgi-associated vesicles from the endoplasmic reticulum to the Golgi apparatus around the centrosome. Similarly the dynein/dynactin motor is the predominant motor that stimulates the localization of the Golgi apparatus at the cell center [17, 25, 26]. The existing model is that dynein 1 mediates the ER-to-Golgi transport and that the perturbation of dynein 1 leads to the failure of the transport of Golgi-associated vesicles to the central Golgi apparatus. In such a case, the newly formed vesicles emerge at the ERES and this results in the Golgi mini-stacks and Golgi scattering phenotype [21, 22]. In addition, the targeted ablation of minus end-directed kinesin-14 KIFC3 was a result of the fragmentation and scattering of the Golgi apparatus throughout the cytoplasm and inhibited the minus end-directed transport of the Golgi apparatus in adrenocortical cells, but only under cholesterol-depleted conditions [18]. Based on our results, we suggest that minus end-directed kinesin-14 KIFC1 also plays a similar role in the inward motility of Golgi-related vesicles and the accumulation the Golgi ribbons around the MTOC (Figure 4A; Supplementary Movies 7, 8). The depletion of KIFC1 results in defects of ER-to-Golgi transport in HEK293T cells. In this case, the new Golgi-associated vesicles may fail to transport to the pericentrosomal region and remain at the ERES. This results in the Golgi scattering phenotype (Figures 3, 4). Further studies are needed to address the molecular mechanism of ER-to-Golgi transport and the Golgi positioning and integrity.

The classical model for molecular motor kinesin and cargo interactions is that the tail domain can recognize and bind to the cargos where the motor domain can then walk along the microtubules using ATP hydrolyzing energy and generate the force required for cargo transport [27]. A recent study revealed a new model for Golgi positioning that the Kinesin-3 KIF1C not only mediates the conventional vesicle transport along the microtubules around the Golgi apparatus, but also statically bound to the Rab6A vesicles to hold vesicles around the Golgi apparatus and contribute to the Golgi structural maintenance via both its motor and tail domain [28]. Surprisingly, in this study we found that the motor domain of the minus end-directed kinesin-14 KIFC1 could also recognize and statically bind to the Golgi apparatus. This interaction between the motor domain of KIFC1 and Golgi apparatus could also abrogate the motor domain-dependent motility. In a manner similar to Kinesin-3 KIF1C, the tail domain of KIFC1 can also bind to the endocytic vesicles or Golgi-associated vesicles. We therefore inferred that the motor domain and tail domain of KIFC1 can simultaneously contribute to the Golgi structural integrity. Moreover, previous studies have revealed that unlike Kinesin-3 KIF1C, which only has one microtubule binding site, kinesin-14 KIFC1 harbors two microtubule binding sites, one at the motor domain and another at the tail domain, which enable kinesin-14 KIFC1 proteins to function as crosslinkers between microtubules [19, 29, 30].

### A new stationary binding model for the Golgi positioning and structural maintenance mediated by kinesin-14 KIFC1

Together, we suggest here a new stationary binding model for the roles of kinesin-14 KIFC1 in Golgi positioning suggesting that the motor domain of KIFC1 can interact and bind to the Golgi apparatus and that the tail domain of KIFC1 is responsible for the static crosslinking of the Golgi apparatus to the microtubules around the centrosomes. In this model, we propose that in the absence of *kifc1* gene, the static crosslinking between the Golgi apparatus and the microtubules around the MTOC is disrupted. Moreover, the minus-end directed transport of the Golgi-associated vesicles to microtubule minus ends mediated by kinesin-14 KIFC1 is also inhibited. These two main reasons can explain why both *kifc1* gene knockdown and knockout in HEK293T cells result in the disorganization of the Golgi apparatus and the dispersal of the Golgi apparatus into cisternae and mini-stacks.

Based on our data, we propose a new stationary binding model for how KIFC1 stimulates the localization of the Golgi apparatus at the microtubule organizing center near the nucleus and how it acts to maintain the well-organized architecture of the Golgi apparatus (Figure 6D). The microtubules are organized at the MTOC near the cell center and form characteristic radial arrays extending toward the cell periphery in non-polarized mammalian cells. In the cytoplasm, kinesin-14 KIFC1 proteins traffic along the microtubules, from the plus ends to the minus ends, and accumulate at the pericentrosomal region. The dynamic movements of kinesin-14 KIFC1 motors along the microtubules are fulfilled by the step-by-step walking of the motor domain using ATP-hydrolyzing energy and the cargo-binding affinity of the tail domain. During vesicle transport, kinesin-14 KIFC1 transports the Golgi-associated vesicles to the minus ends near the MTOC through the minus-end directed motility. Kinesin-14 KIFC1 facilitates the accumulation of the Golgi vesicles at the MTOC to mediate the organization of the Golgi apparatus (Supplementary Figure 5A, 5B).

Moreover, at the minus end of the microtubules around the MTOC, kinesin-14 KIFC1 protein can bind to the Golgi apparatus via its motor domain and can also statically bind to the microtubules via its tail domain. Kinesin-14 KIFC1 servers as a static crosslinker between the Golgi apparatus and the microtubules around the pericentrosomal region near the nucleus. In the non-polarized mammalian cells, loss-of-function of *kifc1* gene leads to the disruption in the central positioning the Golgi apparatus and the dispersal of the Golgi cisternae and mini-stacks throughout the cytoplasm (Supplementary Figure 5A, 5B). Our findings may provide novel insights into the roles of kinesins and the molecular mechanism involved in both the positioning and architectural maintenance of the Golgi complex in non-polarized mammalian cells.

## MATERIALS AND METHODS

### Cell culture and transfection

The following cell lines were used: HEK293T cell (ATCC CRL-3216) and HeLa cell (ATCC CCL-2). Cell lines were cultured in Dulbecco's modified Eagle's medium (DMEM; GIBCO) supplemented with 10% fetal bovine serum (GIBCO) and 100 U/ml penicillin/streptomycin (GIBCO) at 37°C in a 5% CO_2_ humidified atmosphere. For overexpression, plasmids were transfected with Lipofectamine 3000 transfection reagent (Life Technologies) and cells were harvested for subsequent analyses at 24 hr post-transfection.

### Antibodies and reagents

The following antibodies and reagents were used in this study. For immunofluorescence, rabbit monoclonal anti-KIFC1 (1:200, ab172620, Abcam), used in Figures 1, 2, 3, 4, 5 and Supplementary Figures 1–5; rabbit polyclonal anti-KIFC1 (1:200, NB100-40844, Novus Biologicals), used for immunofluorescence only in Supplementary Figure 1G and Figure 6A, 6B; mouse monoclonal anti-GM130 (1:200, 610823, BD Biosciences); rabbit monoclonal anti-γ-tubulin (1:500, ab179503, Abcam); mouse monoclonal anti-α-tubulin (1:500, AT819, Beyotime). Secondary Alexa Fluor 488-conjugated anti-rabbit antibody (1:500, A0423) and Alexa Fluor 555-conjugated anti-mouse antibody (1:500, A0460, Beyotime. DAPI (Beyotime) was used to visualize the nucleus. For western blot, rabbit monoclonal anti-KIFC1 (1:5000, ab172620, Abcam), rabbit polyclonal anti-KIFC1 (1:200, NB100-40844, Novus Biologicals), mouse monoclonal anti-GAPDH (1:2000, D190090, BBI), rabbit polyclonal anti-β-Actin (1:2000, D110001, BBI), mouse monoclonal anti-Flag antibody (1:2000, AF519-1, Beyotime). Secondary goat-anti-rabbit HRP-conjugated antibody (1:2000, A02028, Beyotime), secondary goat-anti-mouse HRP-conjugated antibody (1:2000, D110087, BBI).

### Plasmid construction

The following overexpression and N-Flag tagged plasmid construct was used in this study: pCMV-N-Flag (Beyotime). First, the full-length of EGFP was cloned and inserted in to the backbone of a pCMV-N-Flag vector to generate pCMV-N-Flag-EGFP vectors. Next, full-length KIFC1 (NM_002263.3; 1-673 AAs), KIFC1ΔC (1-310 AAs), KIFC1ΔN (142-673 AAs), KIFC1 Motor (311-673 AAs), KIFC1 Stalk (143-310 AAs), KIFC1 Tail (1-142 AAs), Motor (311-492), Motor (493-673), KIFC1 (42-310), KIFC1 Tail (42-142) were cloned from the HEK293T cDNA, and inserted into the corresponding restriction sites of pCMV-N-Flag-EGFP vector, respectively. The plasmids were selected and sequenced for validation.

### RNAi knockdown

The sequences for shRNA targeting *kifc1* were: *kifc1* shRNA-1, 5′-GGACTTAAAGG GTCAGTTATG-3′; *kifc1* shRNA-2, 5′-GCAAGCTACGT AGAGATCTAC-3′; *kifc1* shRNA-3, 5′-GGTCAGTTATG TGACCTAAAT-3′; *kifc1* shRNA-4, 5′-GCCAACAGG AGCTGAAGAACT-3′; *kifc1* shRNA-5, 5′-GCCCAGAA TGAACGGTCATCA-3′. The sequence of control *Luciferase* shRNA (shLuc) was 5′-GCTTACGCTGAG TACTTCGAA-3′. The corresponding shRNAs were synthesized, annealed and cloned into the backbone pRNAi-U6.2/Lenti virus vector (Biomics). For transient transfection (for 24-well plate), 500 ng shRNA plasmid and 1 μl P3000 enhancer reagent (Life Technologies) were diluted in 50 μl Opti-MEM reduced serum medium (GIBCO). And then 1 μl Lipofectamine 3000 reagent (Life Technologies) was diluted in 50 μl Opti-MEM reduced serum medium. The Lipofectamine 3000 complexes were prepared by combining these two solutions and then incubated in eppendorf tubes for 5 min at 25°C. The transfection components were added directly into the culture medium according to the manufacturer's instructions (Life Technologies). Cells were collected for subsequent analysis at 72 hr or 90 hr after transfection.

### CRISPR-Cas9 system

Target selection for sgRNA. Guide sequence for CRISPR-Cas9 gene targeting was designed using an online CRISPR Design Tool (http://crispr.mit.edu/) according to the standard protocol. The 23bp Cas9-gRNAs ending with one arbitrary nucleic acid and two guanines (NGG, the PAM sequence), which can recognize the target DNA in the coding region of human *kifc1* gene, were designed and selected. The guide sequence for *kifc1* knockout in human HEK293T cell lines were listed as follows: sg*kifc1*-1, 5′-AACTAAAACGGTGCCGTGAG-3′ (for *kifc1^−/−^* clone 1); sg*kifc1*-2, 5′-CCGTGAGAGGACTCAAACGT-3′ (for *kifc1^−/−^* clone 2).

sgRNA expression plasmid construction. The oligo pairs encoding the 20-nt gudie sequence were annealed and ligated into the plasmid pSpCas9 (BB) (Addgene ID: 42230). The CRISPR plasmids were selected and validated by DNA sequencing. Two successful gRNA expression vectors for human *kifc1* gene editing were selected for further experiments.

Clonal isolation of cell lines. Cells were transfected with sgRNA expression plasmids using the Lipofectamine 3000 reagent (Life Technologies) according to the manufactures’ instructions. For transfection (for 24 well-plate), 500 ng sgRNA plasmid, 1 μl P3000 reagent were mixed in 500 μl Opti-MEM medium; 1 μl Lipofectamine 3000 were diluted in 50 μl Lipofectamine 3000 reagent (Life Technologies). These two solutions were mixed and incubated for 5 min at 25°C. The Lipofectamine complexes were added into the culture medium. Cells were then cultured for 48 hr before passaging. And then we selected 96 monoclonal cell lines, and cultured in 96-well plate for clonal isolation. And then these cells were cultured for 25 days and were passaged every 4–5 days.

Functional testing. Successful CRISPR-Cas9 editing cells were confirmed at the polyclonal stage by sequencing. Sequencing primers were designed to amplify the region surrounding the Cas9 targeting site. The primers were listed as follows: Primer target forward 1, 5′-GTGAGAGGCTGGGATAGGGA -3′; Primer target reverse 1, 5′-CCCTCCGTTCTTCCTGCAAT-3′. The PCR products were validated by sequencing to examine the genomic deletion or inversion. The western blot and immunofluorescence experiments were also used to further demonstrate the completely gene knockout in the selected cell lines.

### Western blot analysis

Whole-cell extracts were separated by 10% SDS-PAGE and transferred to PVDF membranes. After being blocked with 5% nonfat milk in 0.1% TBST for 1 hr, the samples were then incubated with the primary antibody for 12 hr at 4°C. The membranes were then incubated with secondary HRP-conjugated antibody and visualized using the chemiluminescent substrate (Thermo). Anti-GAPDH antibody (1:2000, BBI) was used as a loading control,

### Immunofluorescence and confocal microscopy

Cells were fixed at 4% paraformaldehyde in PBS pH7.4 for 10 min at room temperature and then rinsed three times in PBS for 15 min. Cells were then permeabilized with 0.25% Triton X-100 in PBS for 10 min. After blocking at 1% BSA/PBST (containing 0.1% Tween-20) for 1 hr, the cells were incubated in the diluted primary antibody in 1% BSA/PBST for 12 hr at 4°C. Cells were then rinsed in PBS for 15 min, followed by secondary antibody incubation for 1 hr at room temperature. After incubation with DAPI for 5 min, the slides were mounted in the anti-fade mounting medium. The fluorescence images were collected using a confocal laser-scanning microscope (CLSM 710; Carl Zeiss) with a Plan Apochromat 63×/1.4 NA oil objective.

### Time-lapse microscopy and fluorescence recovery after photo-bleaching

Cells were grown on 12×12 mm glass coverslips in 35mm plate (glass bottom; Cellvis; Cat no. D35C4-20-1-N) at 37°C in a 5% CO_2_ humidified atmosphere. At 24 hr post-transfection the cells were immediately collected and imaged on a Zeiss LSM710 microscope equipped with a Plan Apochromat 63×/1.4 NA oil-immersion objective and a 488-nm laser with standard filter sets at 37°C. For time-lapse microscopy, images were captured within 60s at 1s/frame rate. Before the FRAP experiments, the cells were co-stained with the Golgi-Tracker according to the manufacturer's protocols (Beyotime). FRAP experiment were conducted using the strong 405-nm laser for 500 ms to photobleach the fluorescence. FRAP data was monitored for 250 frames at 500 ms/frame. Supplementary Movies were generated using the Zeiss 2009 software at 15 frames/second. FRAP data was analyzed and the fluorescence intensities of EGFP fusion proteins were then quantified using the ImageJ software.

### Image processing and analysis

The immunofluorescence images were acquired by the Zeiss ZEN2009 software (Zeiss). Three dimensional images were taken at 0.41 μm intervals and z-stacks were rendered manually using ZEN2009 software. The gray scale or immunofluorescence intensity of the graphs, including gels and images, were measured and quantified using the Image J software (ImageJ, NIH). All images were prepared using the Photoshop 5.0 software according to the standard instructions.

### Statistical analysis

Data from at least three independent experiments was subjected to GraphPad Prism 6.0 and SPSS20.0 statistical software to test the statistical significance with the appropriate tests. Statistical analyses were performed on mean values of three or more biological replicates using unpaired two-tailed Student's *t*-test. Results were indicated as means ± SEM. All statistical graphs were constructed out using GraphPad Prism 6.0 software. *P* values ≤ 0.05 were considered as statistically significant. ns, not significant; *P < 0.05; **P < 0.01; ***P < 0.001.

## SUPPLEMENTARY MATERIALS FIGURES AND TABLES



















## Abbreviations

FRAP: fluorescence recovery after photobleaching
MTOC: microtubule organizing center
NLS: nuclear localization signal
γ-TuRC: γ-tubulin ring complex
