# Supplementary material for: Minus end-directed kinesin-14 KIFC1 regulates the positioning and architecture of the Golgi apparatus
Source: Oncotarget. 2017 Apr 5;8(22):36469–83. doi: 10.18632/oncotarget.16863 (PMC5482669; doi:10.18632/oncotarget.16863)
Supplement: Supplementary file 1 [file oncotarget-08-36469-s001.pdf]

Minus end-directed kinesin-14 KIFC1 regulates the positioning and architecture of the Golgi apparatus

Supplementary Materials

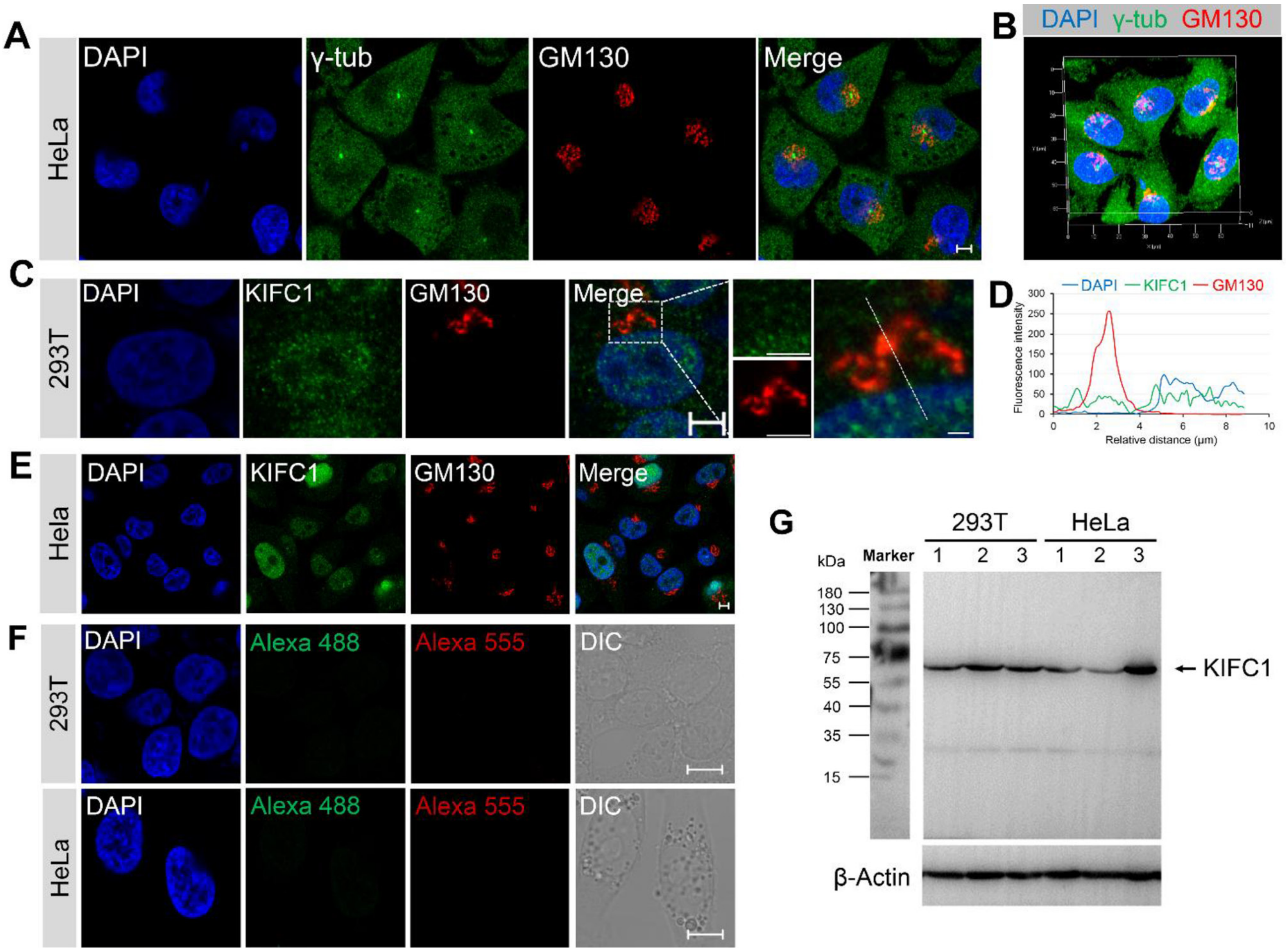

Supplementary Figure 1: Kinesin-14 KIFC1 localized to the Golgi apparatus at cultured HeLa cells. Related to Figure 1. (**A**) Representative confocal images of γ-tubulin and the Golgi marker GM130 in HEK293T cells. DAPI, blue; γ-tubulin, green; GM130, red. Scale bars, 5 μm. (**B**) Three-dimensional image of the fluorescence signals of DAPI (blue), γ-tubulin (green) and GM130 (red) in HeLa cells. (**C**) Representative immunofluorescence images of KIFC1 and GM130 in 293T cells. Zoom images are the enlargement of the indicated boxes. DAPI (blue), KIFC1 (green), GM130 (red). Scale bars, 5 μm. (**D**) The line scan analyses of the fluorescence intensities of DAPI, KIFC1 and GM130 in HeLa cells using the ImageJ software (NIH). A portion of KIFC1 proteins localized at the Golgi apparatus (GM130, the Golgi marker). X axis, relative distance (μm); Y axis, the fluorescence intensity. (**E**) Representative confocal images of KIFC1 and the Golgi marker GM130 in HeLa cells. DAPI, blue; KIFC1, green; GM130, red. Scale bars, 5 μm. (**F**) Immunofluorescence images of control groups without primary antibodies, but incubated with the secondary Alexa 488-or Alexa 555-conjugated antibodies. Scale bars, 10 μm. (**G**) Western Blot of the HEK293T and HeLa cell lysates using KIFC1 antibody. Arrow indicates KIFC1 signals.

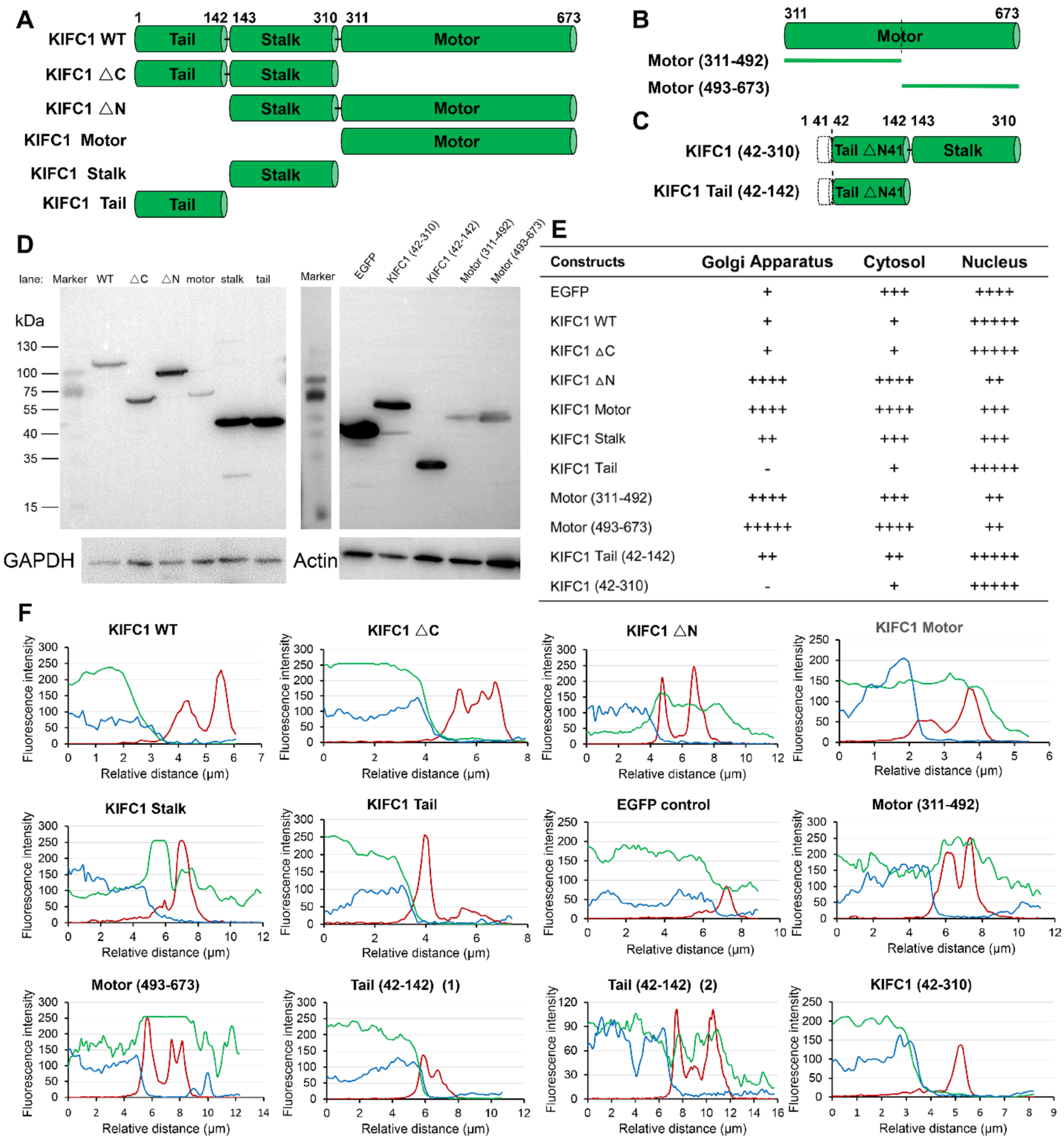

Constructs	Golgi Apparatus	Cytosol	Nucleus
EGFP	+	+++	++++
KIFC1 WT	+	+	+++++
KIFC1 ∆C	+	+	+++++
KIFC1 ∆N	++++	++++	++
KIFC1 Motor	++++	++++	+++
KIFC1 Stalk	++	+++	+++
KIFC1 Tail	-	+	+++++
Motor (311-492)	++++	+++	++
Motor (493-673)	+++++	++++	++
KIFC1 Tail (42-142)	++	++	+++++
KIFC1 (42-310)	-	+	+++++

Supplementary Figure 2: Characterization of KIFC1 EGFP fusion constructs at HEK293T cells. Related to Figure 1. (**A**) Domain organizations of KIFC1 full-length protein (wild type) and the different constructs used in fluorescence microcopy assays. The minus end-directed kinesin-14 KIFC1 protein consists of three functional domains: the N-terminal tail domain, the coiled-coil stalk domain and the C-terminal motor domain. (**B**) Schematic of the KIFC1 motor domain constructs: Motor (311-492) and Motor (493-673). (**C**) Schematic of the KIFC1 ΔN41 constructs (with depletion of the N-terminal 41 amino acids): KIFC1 (42-310) and KIFC1 Tail (42-142). (**D**) Western Blot analysis of the KIFC1 fusion protein constructs using the rabbit anti-Flag antibody. GAPDH (left panel) or β-Actin (right panel) was used as a loading control. The size (kDa) of two marker lines (left and right) are the same. (**E**) Diagram summarizes the distribution patterns of different KIFC1 EGFP fusion proteins at the Golgi apparatus, the cytosol and the nucleus in the HEK293T cells. The line scan analyses of the fluorescence intensities of different KIFC1 EGFP fusion proteins at the Golgi apparatus, the cytosol and the nucleus in HEK293T cells were analyzed and quantified using the ImageJ software (NIH). Approximately 300-500 cells were quantified in each group. "-" indicates no obvious signals; from "+" to "+++++" indicates the gradually increase of the fluorescent signals ("+" sign indicates ~10%;"+++++" sign indicates ~50%). (**F**) Quantitative fluorescence intensity analyses of KIFC1 and other EGFP fusion constructs in HEK293T cells in Figure 1E. DAPI (blue), EGFP fusion proteins (green), GM130 (red). The region is from middle of nucleus to cytoplasm and across the middle of the Golgi apparatus in each transfection group in Figure 1E. The quantification and statistical analyses was done by linescans using ImageJ software.

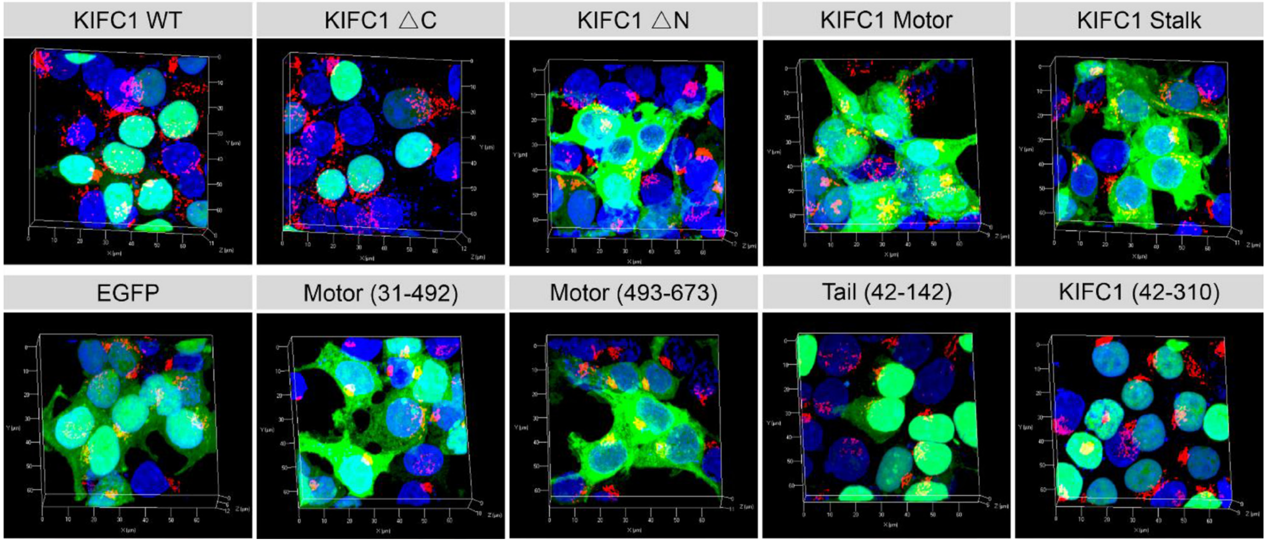

Supplementary Figure 3: Both the KIFC1 motor and tail domain recognize and bind to the Golgi apparatus. Related to Figure 2. Representative three-dimensional images of the fluorescence signals of DAPI (blue), EGFP fusion proteins (green) and GM130 (red) in HEK293T cells. HEK293T cells were transiently transfected with KIFC1 WT (wild type, 1-673), KIFC1 ΔC, KIFC1 ΔN, KIFC1 Motor, KIFC1 Stalk, KIFC1 Tail, EGFP, Motor (311-492), Motor (493-673), Tail (42-142), Tail (42-310), KIFC1 (42-310) for 24 hr.

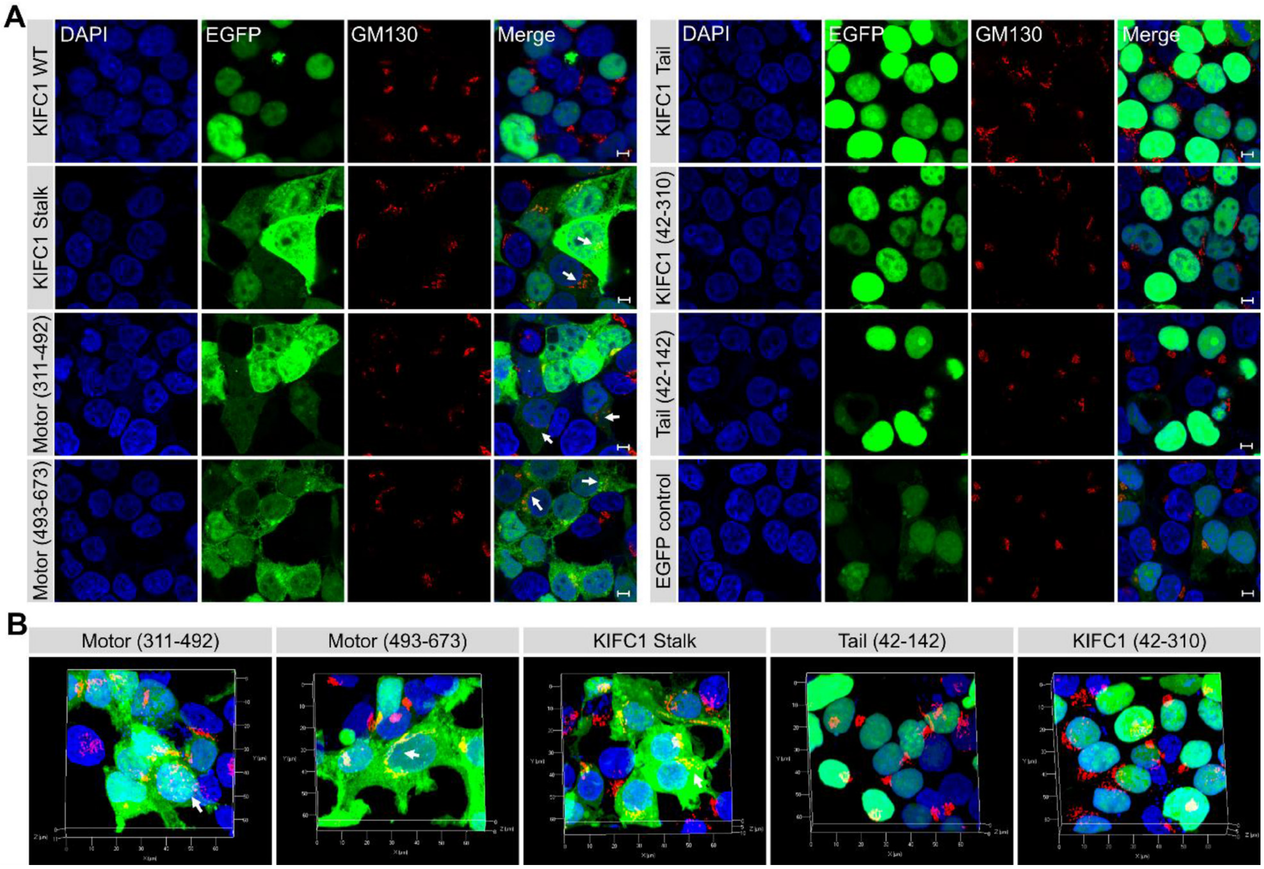

Supplementary Figure 4: Minor effects of other KIFC1 mutant constructs on the Golgi apparatus. Related to Figure 2. (**A**) HEK293T cells were transiently transfected with the KIFC1 WT, KIFC1 Stalk, Motor (311-492), Motor (493-673), KIFC1 Tail, KIFC1 (42-310), Tail (42-142), EGFP control for 24 hr. DAPI, blue; EGFP fusion proteins, green; GM130, red. Scale bars, 5 µm. (**B**) Representative three dimensional images of KIFC1 mutant constructs in HEK293T cells. DAPI (blue), EGFP fusion proteins (green), GM130 (red).

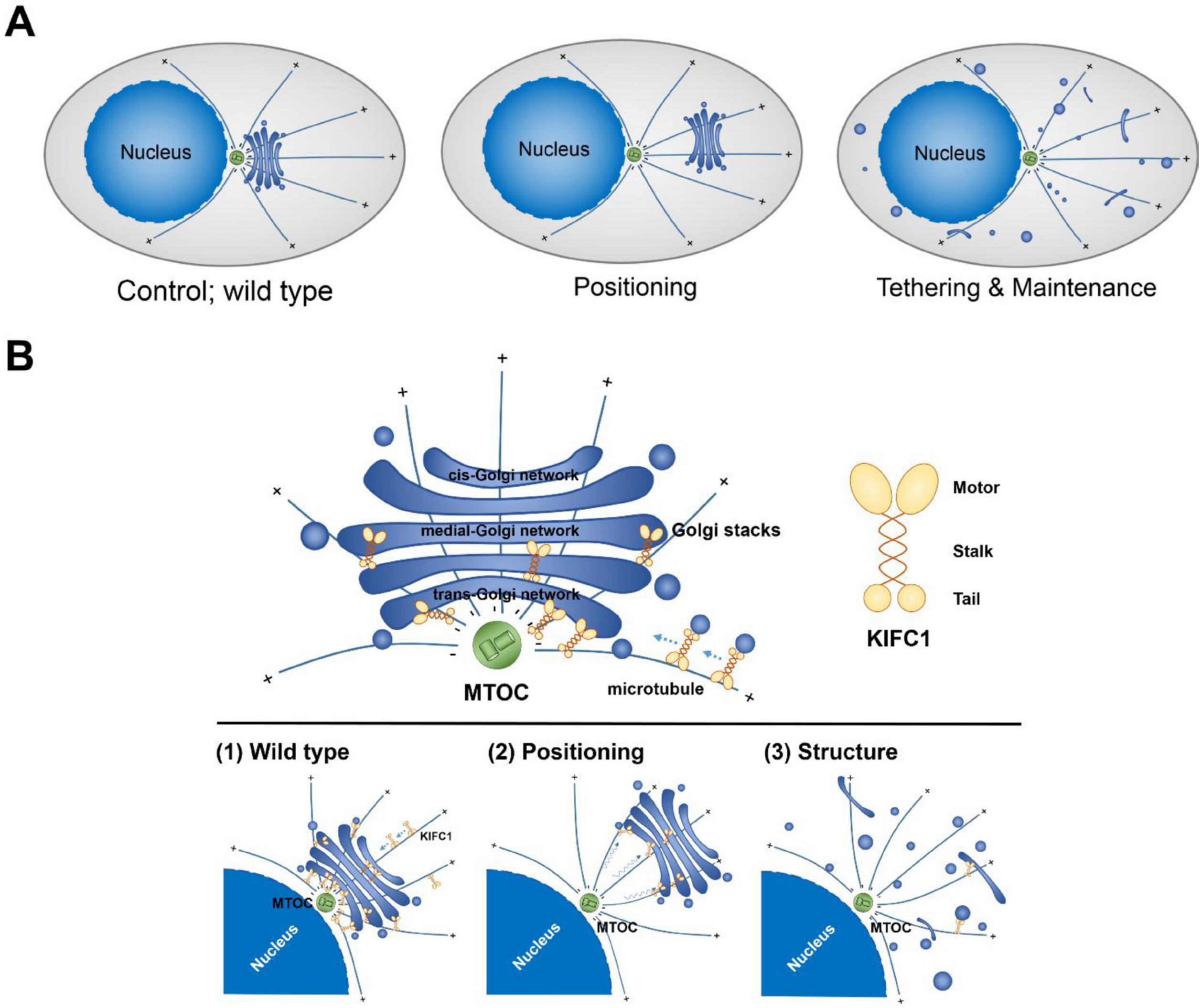

Supplementary Figure 5: Models explaining the results from the kifc1 ablation assays in cultured cells. (**A**, **B**) In control cells (wild type), KIFC1 accumulates at the Golgi apparatus and functions as a crosslinker between the microtubules and the Golgi complex. KIFC1 maintains the architecture of the Golgi apparatus and its localization proximal to the centrosome at the cell center. Minor effects: the structure of the Golgi apparatus is slightly affected, but the Golgi apparatus is drawn away from the nucleus and no longer located at the cell center. Severe effects: the disorganized Golgi apparatus loses the ribbon-like arrangement and the small Golgi cisternae are dispersed throughout the cytoplasm.

Supplementary Movie 1: Time-lapse imaging of KIFC1 WT-EGFP in HEK293T cells. Related to Figure 1. KIFC1 WT-EGFP fusion proteins were shown in green. The images were captured within 60s at 1s/frame rate. This movie is shown at 15 frames/s. See Supplementary_Movie_1

Supplementary Movie 2: Time-lapse imaging of KIFC1 ΔC-EGFP in HEK293T cells. Related to Figure 1. KIFC1 ΔC-EGFP fusion proteins were shown in green. The images were captured within 60s at 1s/frame rate. This movie is shown at 15 frames/s. See Supplementary_Movie_2

Supplementary Movie 3: Time-lapse imaging of KIFC1 ΔN-EGFP in HEK293T cells. Related to Figure 1. KIFC1 ΔN-EGFP fusion proteins were shown in green. The images were captured within 60s at 1s/frame rate. This movie is shown at 15 frames/s. See Supplementary_Movie_3

Supplementary Movie 4: Time-lapse imaging of KIFC1 Motor-EGFP in HEK293T cells. Related to Figure 1. KIFC1 Motor-EGFP fusion proteins were shown in green. The images were captured within 60s at 1s/frame rate. This movie is shown at 15 frames/s. See Supplementary_Movie_4

Supplementary Movie 5: Time-lapse imaging of KIFC1 Stalk-EGFP in HEK293T cells. Related to Figure 1. KIFC1 Stalk-EGFP fusion proteins were shown in green. The images were captured within 60s at 1s/frame rate. This movie is shown at 15 frames/s. See Supplementary_Movie_5

Supplementary Movie 6. Time-lapse imaging of KIFC1 Tail-EGFP in HEK293T cells. Related to Figure 1. KIFC1 Tail-EGFP fusion proteins were shown in green. The images were captured within 60s at 1s/frame rate. This movie is shown at 15 frames/s. See Supplementary_Movie_6

Supplementary Movie 7: Kinetics of KIFC1 Motor-EGFP in HEK293T cell after photobleaching. Related to Figure 5A. KIFC1 Motor-EGFP fusion proteins were shown in green. FRAP data was monitored for 250 frames at 500 ms/frame. This movie is shown at 15 frames/s. See Supplementary_Movie_7

Supplementary Movie 8: Kinetics of KIFC1 ΔN-EGFP in HEK293T cell after photobleaching. Related to Figure 5B. KIFC1 ΔN-EGFP fusion proteins were shown in green. FRAP data was monitored for 250 frames at 500 ms/frame. This movie is shown at 15 frames/s. See Supplementary_Movie_8
